# Effects of voluntary contraction on the soleus H-reflex of different amplitudes in healthy young adults and in the elderly

**DOI:** 10.3389/fnhum.2022.1039242

**Published:** 2022-12-15

**Authors:** Leandra Batista-Ferreira, Natielle Ferreira Rabelo, Gabriel Menezes da Cruz, Juliana Nunes de Almeida Costa, Leonardo Abdala Elias, Rinaldo André Mezzarane

**Affiliations:** ^1^Laboratory of Signal Processing and Motor Control, Faculty of Physical Education, University of Brasília, Brasília, Goiás, Brazil; ^2^Neural Engineering Research Laboratory, Center for Biomedical Engineering, University of Campinas, Campinas, São Paulo, Brazil; ^3^Department of Electronics and Biomedical Engineering, School of Electrical and Computer Engineering, University of Campinas, Campinas, São Paulo, Brazil; ^4^Faculty of Health Sciences, University of Brasília, Brasília, Goiás, Brazil; ^5^Postgraduate Program in Biomedical Engineering, University of Brasília, Brasília, Goiás, Brazil

**Keywords:** recruitment curve, Mmax, spinal cord, humans, computational model

## Abstract

A number of H-reflex studies used a moderate steady voluntary contraction in an attempt to keep the motoneuron pool excitability relatively constant. However, it is not clear whether the voluntary muscle activation itself represents a confounding factor for the elderly, as a few ongoing mechanisms of reflex modulation might be compromised. Further, it is well-known that the amount of either inhibition or facilitation from a given conditioning depends on the size of the test H-reflex. The present study aimed at evaluating the effects of voluntary contraction over a wide range of reflex amplitudes. A significant reflex facilitation during an isometric voluntary contraction of the soleus muscle (15% of the maximal voluntary isometric contraction–MVC) was found for both young adults and the elderly (*p* < 0.05), regardless of their test reflex amplitudes (considering the ascending limb of the H-reflex recruitment curve–RC). No significant difference was detected in the level of reflex facilitation between groups for all the amplitude parameters extracted from the RC. Simulations with a computational model of the motoneuron pool driven by stationary descending commands yielded qualitatively similar amount of reflex facilitation, as compared to human experiments. Both the experimental and modeling results suggest that possible age-related differences in spinal cord mechanisms do not significantly influence the reflex modulation during a moderate voluntary muscle activation. Therefore, a background voluntary contraction of the ankle extensors (e.g., similar to the one necessary to maintain upright stance) can be used in experiments designed to compare the RCs of both populations. Finally, in an attempt to elucidate the controversy around changes in the direct motor response (M-wave) during contraction, the maximum M-wave (Mmax) was compared between groups and conditions. It was found that the Mmax significantly increases (*p* < 0.05) during contraction and decreases (*p* < 0.05) with age arguably due to muscle fiber shortening and motoneuron loss, respectively.

## Introduction

The H-reflex amplitude varies linearly within a limited range of muscle activation in isometric conditions (Hagbarth, 1962; Gottlieb and Agarwal, 1971; Burke et al., 1989; Rüegg et al., 1990; Pierrot-Deseilligny, 1997; Burke, 2016), a phenomenon known as “automatic gain compensation” (Matthews, 1986). The simplest physiological explanation for this reflex modulation is that the motoneurons (MNs) not recruited during rest will have their membrane depolarized by the descending drive, increasing the probability to fire an action potential from the afferent volley. However, there are several spinal cord mechanisms contributing toward reflex modulation during voluntary contraction, such as reduced presynaptic inhibition (PSI) at Ia terminals (Hultborn et al., 1987; Iles and Roberts, 1987), decreased Ib inhibition (Marchand-Pauvert et al., 2002), increased firing rate of afferent Ia due to alpha-gamma co-activation (Vallbo, 1974; Gottlieb and Agarwal, 1978; Burke et al., 1979), reduced homosynaptic depression (Burke et al., 1989), and reduced excitability of Renshaw cells (Hultborn and Pierrot-Deseilligny, 1979). Therefore, changes in reflex excitability during background muscle activity is probably multifactorial, and one or more of those physiological mechanisms might be compromised to some extent along the aging process (Brooke et al., 1989; Kido et al., 2004).

There is some inconsistency in the literature on the effects of contraction upon reflex excitability in the elderly (Angulo-Kinzler et al., 1998; Kawashima et al., 2004; Obata et al., 2010; Klass et al., 2011). The conflicting results might be related to different methodological approaches used in those researches, such as the neurophysiological probe (electrically evoked H-reflex or different latency components of the stretch reflex), target muscle (ankle flexors or extensors that respond differentially to stretch), posture (standing, prone, and sitting position) and different amplitudes of the test H-reflex. Of note, the effects of voluntary muscle contraction on reflex excitability in the elderly have been shown mostly for a fixed amplitude of test reflex (e.g., the maximal reflex response–Hmax) (Kawashima et al., 2004; Klass et al., 2011). Despite previous studies on the H-reflex recruitment curve (RC), the reflex responses of intermediated sizes (lower than Hmax) have not yet been fully explored, i.e., specific parts of the RC have never been examined in detail. This is an interesting point as numerous interventions were shown to differentially affect the amplitude parameters extracted from the RC (Dragert and Zehr, 2011; Vila-Chã et al., 2012; Quadrado et al., 2020; Magalhães et al., 2021). Thus, it is worth examining critical points of the ascending limb of the RC by, e.g., a sigmoid fit (Klimstra and Zehr, 2008) to address possible differential effects of contraction on reflex modulation of the soleus muscle, which is one of the main postural muscles responsible to maintain balance during upright stance (Mezzarane and Kohn, 2007).

The maintenance of a background activity at 10–30% of the maximal voluntary isometric contraction (MVC) has been widely employed, mainly, to hold the motoneuron pool excitability and to reduce H-reflex variability (Marchand-Pauvert et al., 2002; Zehr, 2002; Clark et al., 2007; Chen et al., 2010; Mezzarane et al., 2017). This procedure is also relevant to match the amount of muscle activation in upright stance as well as to prevent the effects of fatigue due to a relatively weak contraction (Earles et al., 2001; Kido et al., 2004; Klass et al., 2011; Baudry and Duchateau, 2012). Moreover, the H-reflex has higher reliability at intensities of contraction below 30% MVC (Chen et al., 2010). Finally, it has been reported that the relation between muscle activation and H-reflex amplitude is nearly linear for relatively low background electromyogram (EMG) activity in both young adults (below 50% MVC) (Butler et al., 1993; Löscher et al., 1996) and the elderly (up to 30% MVC) (Klass et al., 2011). Indeed, the amount of H-reflex facilitation of an ankle dorsiflexor was not different between young adults and the elderly up to 30% of the MVC, and this might be an indicative that presynaptic mechanisms significantly interfere with reflex modulation only at higher intensity of muscle activation (above 30% MVC) in the elderly (Klass et al., 2011).

These observations raise the question about the relevance of spinal cord mechanisms in the modulation of reflex responses during relatively low levels of voluntary contraction (e.g., enough to maintain an upright posture considering an ankle plantar flexor). One may suggest that the descending drive is dominant in modulating reflex excitability during a constant moderate muscle activation. In this regard, it would be desirable to assess the contribution of the descending drive alone to spinal cord excitability by, for example, qualitatively comparing the parameters of the H-reflex RC from humans to the parameters obtained from the RC generated by a computer model of the spinal cord (Cisi and Kohn, 2008; Elias et al., 2012; Nakajima et al., 2017). The model from Cisi and Kohn (2008) comprises the soleus motoneuron pool responses to varying afferent input with a constant cortical drive, and is appropriate to simulate the two experimental conditions: rest and voluntary contraction below 30% MVC (e.g., at 15% MVC; Kido et al., 2004).

To our knowledge, no study has systematically evaluated age-related differences of the H-reflexes of different sizes under an intermediated level of voluntary contraction. We hypothesized that, during a moderate soleus muscle activity (15% MVC), the amount of reflex facilitation will not differ between both groups for the H-reflex amplitudes from the ascending limb of the RC. This might imply that any involvement of spinal cord mechanism in the elderly will not significantly interfere with reflex modulation, regardless of its size. Hence, the descending drive alone would be sufficient (or predominant) to modulate reflex responses. If that is true, differential effects of a moderate contraction between both groups could be ruled out in investigations about the influence of aging processes on the modulation of reflexes with different amplitudes.

Lastly, there is no agreement in the literature regarding changes in the maximal direct response amplitude (Mmax) in different states of muscle activation. There are reports showing either an increase (Nagata and Christianson, 1995; Frigon et al., 2007; Racinais et al., 2013) or absence of changes (Duclay et al., 2008; Grosprêtre and Martin, 2012) in Mmax during contraction. Similar discrepancy was noticed in the comparison of Mmax between the elderly and young adults, with either a decrease in the elderly (Chalmers and Knutzen, 2002; Scaglioni et al., 2002, 2003) or absence of changes in Mmax (Kawashima et al., 2004; Nakazawa et al., 2006; Piirainen et al., 2012). Therefore, it appears worthwhile to reevaluate both the age-related changes in Mmax amplitude and its dependence on voluntary muscle activation.

The present work aimed at: (1) assessing the effects of a moderate voluntary contraction on reflex modulation for the ascending limb of the soleus RC in both groups (young and elderly); (2) comparing experimental results with a model of the spinal cord to reinforce the hypothesis that the descending drive is a dominant factor in modulating the reflex pathway; (3) evaluating the changes in Mmax in different conditions (rest and contraction) and between groups.

## Materials and methods

### Participants

Twenty-nine participants were divided in two groups. One group was composed of 15 young adults (Young) and the other was composed of 14 elderly community-dwelling older adults (Elderly) (Table 1). All participants gave their written consent approved by a local ethics committee according to the declaration of Helsinki. They were submitted to an anamnesis to assess their physical and mental skills (Folstein et al., 1975) and were told to refrain from any kind of stimulating substances 24 h prior the tests. Participants with orthopedic problems or any other neurodegenerative diseases were excluded from the study. The eligible elderly participants reported no pre-existing neurological diseases such as Parkinson’s disease, dementia, cardiovascular disease, arthritis, and any stroke event. They were able to hear, to communicate verbally, as well as to understand the instructions and procedures.

**TABLE 1:** Descriptive statistics of the participants.

	Genders		Age (years)		Height (m)		Weight (kg)		BMI (kg/m^2^)	
Participants	M	F	mean	SD	mean	SD	mean	SD	mean	SD
Young (*n* = 15)	10	5	24.0	4.4	1.71	0.1	64.8	6.8	22.4	3.6
Elderly (*n* = 14)	3	11	68.9	5.1	1.65	0.1	67.4	6.2	24.8	2.3

n = number of participants; M = male; F = Female; SD = Standard Deviation; m = meters; kg = kilograms; BMI = Body Mass Index.

### Data acquisition

Surface electrodes (Ag/AgCl, 0.8 cm diameter) were positioned 2 cm below the two heads of the gastrocnemius muscle, above the soleus aponeurosis with 2 cm distance between each electrode. The ground electrode was positioned on the styloid process. The EMG were amplified and filtered (10 to 1 kHz) by a MEB-2300K system (Nihon-Kohden, Japan) and acquired at 5 kHz using a 16-bit data acquisition system (USB 6363, National Instruments, EUA). The EMG window had 100 ms duration with a 30 ms pre-stimulus period. All data were stored for posterior analysis using custom programs written in Matlab (Mathworks, Natick, MA, USA).

### Procedures

The H-reflexes were elicited in the soleus muscle by a percutaneous electrical pulse (1 ms duration) applied to the posterior tibial nerve at the popliteal fossa by one of the four stimulation channels of the MEB-2300K system (each one with maximal output of 100 mA and resolution of 0.1 mA). The participants were comfortably seated in a specially designed structure with hips and ankles at 90^°^ and the knees flexed at 110^°^. They were tested in two different conditions: at rest and with 15% of MVC of plantar flexors (active condition). The MVC was defined as the highest force exerted during the performance of a maximum voluntary contraction of ankle extensors along 5 s, and the root mean square (RMS) value evaluated from the soleus EMG (in steps of 100 ms) was used as a reference for the maximal voluntary contraction (Nakajima et al., 2014; Suzuki et al., 2014; Mezzarane et al., 2017).

Both, the lowest intensity of current to elicit the threshold H-reflex (Hth), and the intensity to evoke the maximal reflex amplitude (Hmax) were found. Then, the interval between these two intensities was divided in 10 equally spaced values to obtain the ascending limb of the recruitment curve. Since the H-reflex varies even maintaining the same stimulus intensities, the stimuli were delivered five times for each of the 10 intensities previously defined. The intensity of the currents was then increased gradually to evoke the maximum amplitude of the M-wave. To obtain the recruitment curve in the active condition, the same procedure was repeated during a voluntary contraction of the plantar flexors at 15% MVC with the aid of visual feedback of the RMS evaluated from the EMG. As soon as the participant reached the 15% MVC an H-reflex was evoked. This procedure was repeated 5 times for each intensity with 10 s interval between the stimuli with the subject at rest.

### Computer simulations

Computer simulations were performed using a web-based multi-scale neuromuscular simulator representing part of the neuromuscular system that controls leg muscles (Cisi and Kohn, 2008; Elias et al., 2012). For simplification, only the soleus muscle was considered in the simulations. Briefly, the soleus motor nucleus encompasses 900 type-specified two-compartment MN models (800 S-type, 50 FR-type, and 50 FF-type) and 400 Ia afferents (Gillespie et al., 1987; McComas, 1991). The ranges of thresholds to electrical stimulation for motor axons and Ia afferents are presented in Table 2. The synapses between Ia afferents and MNs had a 90% connectivity and 300 nS conductance. The descending volitional motor drive was represented by 400 stochastic inputs to the MN pool with 30% connectivity and 600 nS synaptic conductance. Each motor unit generates both electrical (motor unit action potentials–MUAPs) and mechanical (force twitches) responses. The outputs of the model are the EMG, expressed as the sum of all MUAPs, and the muscle force, as the sum of all muscle twitches. A more detailed description of the mathematical models implemented in the simulator may be found elsewhere (Cisi and Kohn, 2008; Elias et al., 2012).

**TABLE 2:** Range of thresholds to electrical stimulation for motor and sensory axons represented in the computational model of the neuromuscular system.

	Type of MN	Range (mA)
Motor axons	S	12.4–18
	FR	12.2–12.4
	FF	12–12.2
Sensory axons	Ia afferents	9–18

The simulated MVC was defined by setting the activity of descending commands so that the entire MN pool is recruited, and the average discharge rate of the pool is about 20 Hz (Watanabe et al., 2013). The maximal muscle force output achieved in the latter condition was the MVC. Once the MVC was defined, the interspike intervals (ISIs) of descending commands were set to represent two submaximal conditions: at rest (i.e., without spiking activity of the MN pool), and at 15% of the MVC (as measured by the force output).

The stimulation protocol was defined in the model to match the experimental procedures, i.e., a short current pulse (1 ms) was applied to the posterior tibial nerve in a point equivalent to the popliteal fossa (0.66 m from the spinal cord and 0.14 m to the muscle endplate). The recruitment curve of the H-reflex was obtained by adjusting the stimulus amplitude from 10 to 22 mA, with 0.50 mA step. The total simulation time was 2 s, with the electrical stimulus applied after 1 s to reach the model’s steady state. Five simulations were performed for each stimulus intensity.

### Data analysis

H-reflex peak-to-peak amplitude values obtained in all conditions were normalized by Mmax for both groups. The H-reflex and M-wave RCs at rest and with contraction were fit with a sigmoid curve using a general least square model (Klimstra and Zehr, 2008). The main parameters from the curve were the slope of the regression line of the ascending limb of the H-reflex (Hslp) and M-wave (Mslp) curves, H threshold (Hth), H “at” threshold (H@th, found on the fitted RC during contraction using as reference the current that evoked the Hth at rest: Curr Hth), H50 (same amplitude as 50% Hmax), H@50 (found using the same procedure for H@th, but referenced to Curr H50), H100 (reference amplitude representing approximately Hmax), and H@100 (referenced to Curr H100) (Magalhães et al., 2021). The slope of the ascending limb of the H-reflex RC was referenced to the slope of the M-wave to obtain the parameter (Hslp/Mslp). The Hmax was calculated as the average of the 5 highest H-reflex amplitudes. The analysis of simulated data followed the same procedure of the experimental counterparts.

### Statistical analysis

Data were presented as mean ± standard error and tested for normal distribution using the Shapiro-Wilk test. The parameters Hth (rest) and H@th (contraction), H50 and H@50, H100 and H@100, Hslp/Mslp, Hmax and Mmax were compared for both groups with 95% confidence interval (CI). A two-way ANOVA with repeated measures was used to detect possible differences and interactions between type of condition Contraction (Cont.) X No Contraction (Rest) and age group of the participant (Young X Elderly). The level of significance was set at *p* < 0.05.

## Results

All data showed normal distribution. The repeated measures two-way ANOVA revealed that voluntary contraction increased all H-reflex amplitude parameters for both groups, Hth [*f*_(1_,_28)_ = 22.52, *p* < 0.001, *η*^2^ = 0.455], H50 [*f*_(1_,_29)_ = 25.65, *p* < 0.001, *η*^2^ = 0.487], H100 [*f*_(1_,_29)_ = 10.84, *p* = 0.003, *η*^2^ = 0.286], Hmax [*f*_(1_,_29)_ = 8.99, *p* = 0.006, *η*^2^ = 0.257].

**FIGURE 1:**
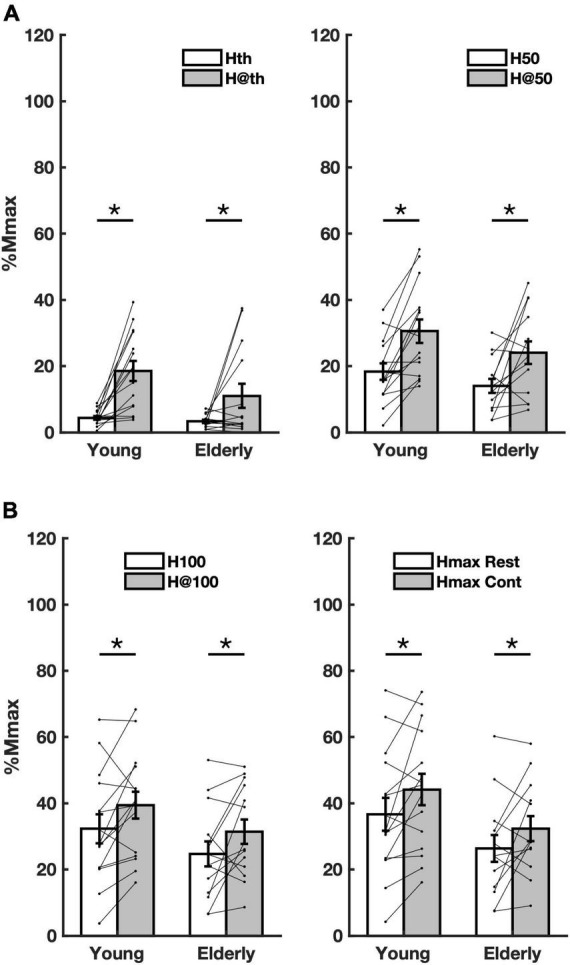
Overall averaged amplitude parameters from both groups in both conditions. **(A)** amplitude parameters Hth (rest), H@th (contraction), H50 (rest), and H@50 (contraction) extracted from the recruitment curve (RC) sigmoidal fit; **(B)** the same as in panel A for the parameters H100, H@100, and Hmax at rest and during contraction. Asterisks mean significant difference (*p* < 0.05) between conditions rest (Rest) and contraction (Cont).

The percent of increase for each parameter and group were: Hth (young = 324.7%, CI = 114.2–535.2); elderly = 230.1% CI = −96.1 to 556.3); H50 (young = 66.8% CI = 44.5–89.0; elderly = 71.7% CI = 40.5–102.9) (Figure 1A); H100 (young = 22% CI = 18.8–25.3; elderly = 27.3% CI = 26.5–28.2); Hmax (young = 20.4% CI = 18.4–22.5); elderly = 22.8% CI = 19.0–26.6) (Figure 1B; Table 3). Furthermore, no differences in H-reflex amplitude parameters between groups were detected to Hth [*f*_(1_,_28)_ = 2.961, *p* = 0.097, *η*^2^ = 0.099]; H50 [*f*_(1_,_29)_ = 2.308, *p* = 0.140, *η*^2^ = 0.079]; H100 [*f*_(1_,_29)_ = 2.20, *p* = 0.149, *η*^2^ = 0.075]; and Hmax [*f*_(1_,_28)_ = 3.36, *p* = 0.078, *η*^2^ = 0.114]. The amount of reflex facilitation was not significantly different between young and the elderly, i.e., no significant interaction was detected between the factors condition and group for Hth [*f*_(1_,_28)_ = 1.988, *p* = 0.170, *η*^2^ = 0.069], H50 [*f*_(1_,_29)_ = 0.246, *p* = 0.624, *η*^2^ = 0.009], H100 [*f*_(1_,_28)_ = 0.007, *p* = 0.932, *η*^2^ = 0.000], and Hmax [*f*_(1_,_28)_ = 0.108, *p* = 0.745, *η*^2^ = 0.004] (Table 3).

**TABLE 3:** Recruitment curve (RC) parameters from experiments with young, elderly and simulations, as well as the respective percentual increases with contraction.

	Simulated (%Mmax)			Young (*n* = 15) (%Mmax)				Elderly (*n* = 14) (%Mmax)			
Amplitude parameters	Rest	Cont.	%	Rest	Cont.	%	CI = %95	Rest	Cont.	%	CI = %95
Hth	5.2	13.4	156	4.37	18.5	324.7	(114.2–535.2)	3.1	11	230.1	(−96.1 to 556.3)
H50	21.9	38.4	74.9	18.3	30.5	66.8	(44.5–89.0)	14	24.1	71.7	(40.5 to 102.9)
H100	38.7	49.5	27.9	32.3	39.4	22	(18.8–25.3)	24.7	31.4	27.3	(26.5 to 28.2)
Hmax	43.9	51.5	17.3	36.7	44.1	20.4	(18.4–22.5)	26.3	32.4	22.8	(19 to 26.6)

Rest= no contraction; Cont= with contraction; % = percentage of increase; CI= confidence interval.

**FIGURE 2:**
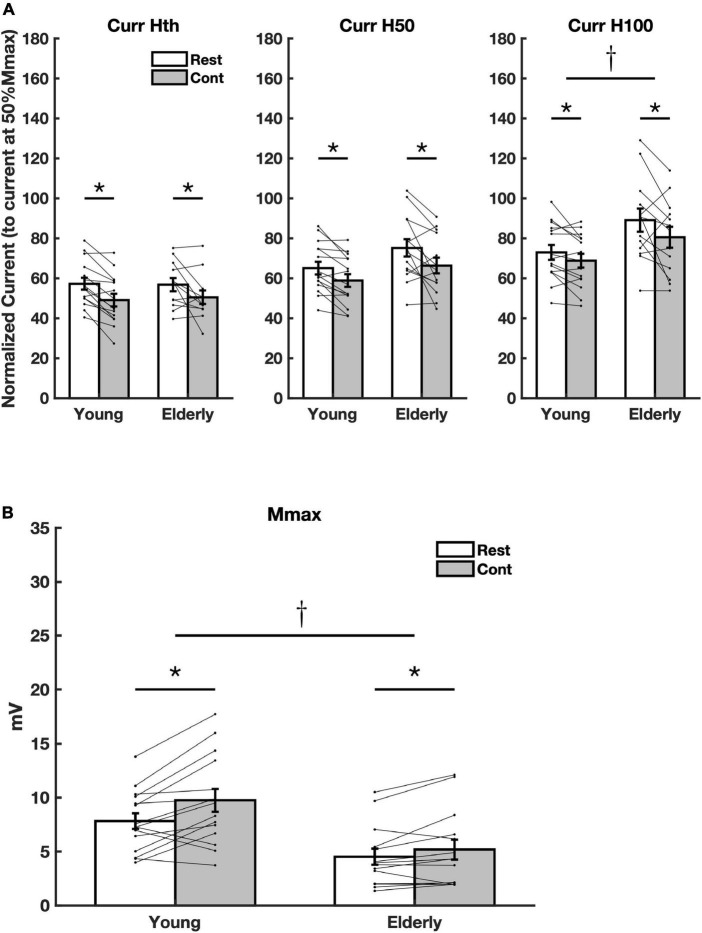
Averaged current values and Mmax. **(A)** Current values related to the amplitude parameters Hth (Curr Hth), H50 (Curr H50), and H100 (Curr H100) extracted from the recruitment curve (RC) sigmoid fit. **(B)** Averaged Mmax amplitude values for all subjects. Asterisks mean significant difference (*p* < 0.05) between conditions (Rest and Cont). The dagger means significant difference between group (Young and Elderly) (*p* < 0.05).

The stimulus intensities (current) related to the parameters Hth, H50, and H100 have shown a significant reduction during voluntary contraction for the Curr Hth [*f*_(1_,_25)_ = 20.28, *p* < 0.001, *η*^2^ = 0.448], Curr H50 [*f*_(1_,_27)_ = 17.64, *p* < 0.001, *η*^2^ = 0.395], and Curr H100 [*f*_(1_,_26)_ = 8.91, *p* = 0.006, *η*^2^ = 0.255] for both groups (Figure 2A). This means that the threshold to evoke H-reflexes of different amplitudes (at the ascending limb of the RC) were reduced during contraction. The amounts of the reduction for each parameter were Curr Hth (young = 14.3% CI = 18.7–9.9, elderly = 11.2% CI = 11.6–10.7), Curr H50 (young = 9.5% CI = 9.1–9.9; elderly = 11.7% CI = 6.6–16.9), and Curr H100 (young = 5.7% CI = 2.9–8.5; elderly = 10.2% CI = 4.7–14.5). No significant difference on the current parameters between groups was detected considering Curr Hth [*f*_(1_,_25)_ = 0.02, *p* = 0.903; *η*^2^ = 0.001] and Curr H50 [*f*_(1_,_27)_ = 3.20, *p* = 0.085, *η*^2^ = 0.106]. However, there was a significant increase in parameter Curr H100 for the elderly [*f*_(1_,_26)_ = 5.35, *p* = 0.029, *η*^2^ = 0.171] (Figure 2A). This means that the thresholds to elicit the higher amplitude H-reflexes increased for the elderly.

No significant effect was detected for the ratio Hslp/Mslp, indicating no meaningful changes in the slope of the ascending limb of the RC during contraction for both groups [*f*_(1_,_25)_ = 2.21, *p* = 0,149, *η*^2^ = 0,081], as well as between groups [*f*_(1_,_25)_ = 2.61, *p* = 0,119, *η*^2^ = 0,094]. On the other hand, a significant increase in Mmax amplitude with contraction was found for both groups (Elderly and Young) [*f*_(1_,_27)_ = 15.51, *p* = 0.001, *η*^2^ = 0.365], while a significant reduction in amplitude for the elderly was observed [*f*_(1_,_27)_ = 10.58, *p* = 0.003, *η*^2^ = 0.282]. However, no interaction between the factors age and voluntary muscle activation was detected [*f*_(1_,_29)_ = 3.78, *p* = 0.062, *η*^2^ = 0.123] (Figure 2B).

**FIGURE 3:**
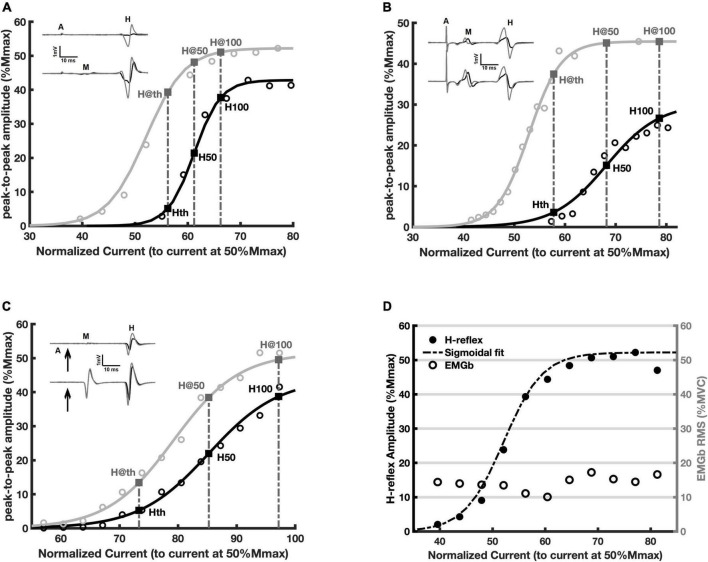
Recruitment curves (RCs) and the respective amplitude parameters obtained at rest (black) and during voluntary contraction (gray). Each circle is the averaged value of five measurements (see Methods). The continuous lines are the fitted curve. **(A)** Representative adult young participant; **(B)** representative elderly participant; **(C)** data from the simulation; **(D)** the same data as in panel **(A)** (filled black circles are the peak-to-peak H-reflex amplitudes), along with the respective EMGb (open circles).

Sigmoid fit to the RCs from representative participants are showed in Figure 3. Each point presented in the Figures 3A–C is an average of 5 H-reflex amplitudes (the computational model produced a RC based on 5 simulation runs). A clear facilitation of the H-reflex is evident for the whole ascending limb of the RC in all cases, the experimental data from young (Figure 3A), the elderly (Figure 3B), and the simulated data (Figure 3C). It is possible to observe a consistent increase in amplitude during contraction as compared to the rest condition: compare the traces and points in black (rest) with those in gray (contraction). The same parameters from the curve fitting to the experimental data were also estimated for the simulated data and are presented in Table 3. The inset in each panel of Figure 3 shows a sweep depicting the M wave and the H-reflex obtained at rest and during contraction. These data referred to the points of RC nearest to H50 (rest) and H@50 (contraction), as well as H100 and H@100 (bottom traces). Figure 3D shows the RC obtained during contraction (same data from Figure 3A) along with the background EMG (EMGb) (open circles). Note that the EMGb is constant at around 15% MVC for all intensities and amplitude values of the H-reflex. This evidenced that changes in H-reflex amplitude were not correlated to the EMGb.

**FIGURE 4:**
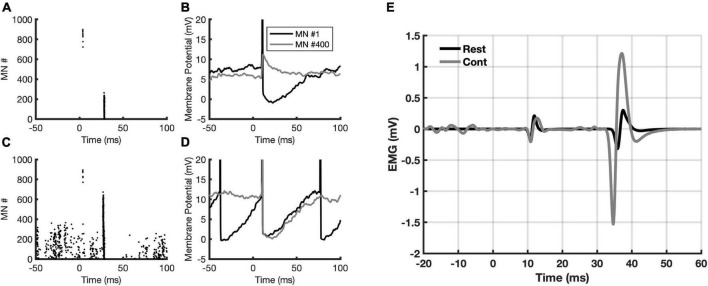
Data from the simulations. **(A)** Recruited motoneurons (MNs) from the stimulation to the posterior tibial nerve; **(B)** the membrane potential from two MNs (#1 and #400) in response to stimulation; **(C)** the same as in panel A, but now with a descending drive at 15% MVC.; **(D)** the same as in panel **(B)**, but now with a descending drive at 15% MVC; **(E)** simulated M-wave and H-reflex at rest (black sweep) and during 15% MVC contraction (gray sweep). Note the EMGb in the gray sweep. The zero in the abscissa indicates the moment when the stimulus is delivered.

Figures 4A, B show, respectively, the number of MNs recruited to compose both the M wave and the H-reflex (depicted in Figure 4E with a black trace), as well as the corresponding membrane potential from two of those MNs (#1 and #400). Figures 4C, D follow the same description as A and B, but during contraction. It is noticeable the increased recruitment of MNs (population coding) in Figure 4C and an increase in the firing rate of both MNs (rate coding) in Figure 4D. During contraction, the number of MNs recruited to compose the M wave (motor axons directly activated by the stimulus) is almost the same, however, the recruitment is higher for the H-reflex due to the descending command. By inspecting the membrane potential of the same MN (e.g., MN #400), we observed that at rest the afferent volley produced a compound excitatory post-synaptic potential. It is also interesting to note that during contraction the MNs are not only recruited by the stimulus, but they are activated by the descending drive as well (observe the MNs recruited before the stimulus being delivered, at negative times in the abscissa of Figure 4C). Those MNs are responsible for the EMGb in Figure 4E (trace in gray), corresponding to 15% of the MVC. The corresponding M wave and H-reflex evoked during contraction are also depicted in Figure 4E (trace in gray).

## Discussion

It is well-known that increments in H-reflex amplitude are evident with increased voluntary muscle activation (Hagbarth, 1962; Gottlieb and Agarwal, 1971; Matthews, 1986; Burke et al., 1989; Rüegg et al., 1990; Pierrot-Deseilligny, 1997; Frigon et al., 2007). The present experimental results corroborate and extend previous findings, i.e., the effects of a contraction at 15% MVC were significant for all amplitude parameters extracted from the ascending limb of the RC, and the absence of interaction (group versus condition) indicates that the strength of these effects did not differ between young and the elderly. The experimental results were qualitatively similar to the simulated data from a biological plausible computational model of the spinal cord with a stationary descending drive, suggesting that intrinsic spinal cord neuronal circuitries acting on reflex modulation have a relatively reduced relevance during a moderate contraction. Finally, in order to clarify the question around the changes in Mmax during contraction, it was confirmed a reduction in the amplitude for the senior group and an increase for both groups as compared to the rest condition.

### Reflex excitability during contraction

The modulation of the H-reflex to some conditionings can be variable according to the amplitude of the test reflex. For instance, only H-reflexes with small amplitudes, at around the threshold for a reflex response, increased their excitabilities following either resistance training of a remote muscle group (Dragert and Zehr, 2011) or induced fatigue of the antagonist muscles around the ankle joint (Magalhães et al., 2021). On the other hand, there are reports on the modulation of high amplitude H-reflexes in response to either endurance training (Vila-Chã et al., 2012) or ischemic pre-conditioning of the contralateral leg (Quadrado et al., 2020). Thus, it is interesting to evaluate the sensitivity of H-reflexes with different amplitudes to voluntary contraction, since some effects might be specific for different recruited MNs.

An inevitable consequence of the aging process is the impairment of neuromuscular system. For example, there is evidence of a reduction in the number of high-threshold MNs and the occurrence of axonal sprouting from the slow motor units (Kadhiresan et al., 1996). This motor unit remodeling was suggested to affect the H-reflex amplitude (Baudry et al., 2015). It has also been reported a compression of thresholds toward motor units of lower forces with age, that arguably represents an adaptive strategy to counterbalance the decreased motor units firing rate (Girts et al., 2020). However, the soleus muscle is composed mainly of low-threshold (slow) MNs (nearly 80%) (Gillespie et al., 1987), thus, it is likely that the thresholds of these cells have some level of “compression” across the whole pool regardless of the age (i.e., a more pronounced homogeneity), which could mitigate to some extent any possible differences of the soleus H-reflex amplitudes between young and older adults.

The diminished current to evoke the reflexes throughout the ascending limb of the RC during contraction, evidenced by the shift of the RCs to the left in both groups (Figure 2A), means that the threshold to evoke a reflex response, regardless of its size, was significantly reduced. Interestingly, the current to elicit high amplitude H-reflexes (Hmax) was augmented in the elderly as compared to young. This is in agreement with the data from Sabbahi and Sedgwick (1982) who reported increased threshold for eliciting H-reflex of high amplitude (70% Hmax) in the elderly. This occurred specifically for H-reflexes with higher amplitudes, since the present results did not reveal significant effect considering the inputs related to H-reflexes with lower amplitudes (for Curr Hth and Curr H50) (Figure 2A). These observations might indicate a more gradual motoneuron recruitment with age. Results from rodents showed age-associated changes in biophysical properties of the MNs (such as lower rheobase and higher input resistance), which reduces the threshold for synaptic input (Kalmar et al., 2009). These biophysical characteristics of MNs do not explain the current results. In this sense, the increased threshold for higher amplitude H-reflexes currently observed is probably related to changes in the sensitivity of the sensory axons to external stimuli (perhaps, related to the degeneration of large diameter nerve fibers leading to an increased activation threshold; Sabbahi and Sedgwick, 1982). Further investigations are necessary to either confirm or refute this possibility as well as to disclose the exact mechanism.

The present modeling results showed that, during voluntary contraction, the increased reflex excitability was qualitatively similar to the experimental results for most of the amplitude parameters extracted from the RC of the soleus muscle. A significant amount of facilitation for the ascending limb of the RC in both groups suggests a similar pattern of MN activation from descending command. However, the computational model comprises the descending drive from cortex and does not contemplate changes in the local modulatory spinal cord networks that would also influence the excitability of the reflex pathway. For instance, age-related alterations in PSI were not included in the model. It is well-established that the level of PSI is reduced during voluntary contraction (Hultborn et al., 1987; Iles and Roberts, 1987; Pierrot-Deseilligny, 1997), but this reduction is significantly diminished in the elderly as compared to young adults (Butchart et al., 1993; Earles et al., 2001). This fact could render a disrupted reflex modulation during contraction for the elderly group.

Nonetheless, the current experimental results showed no significant differences in the amount of reflex facilitation during contraction between young and older adults considering all the amplitude parameters extracted from the RC (representing the low, intermediated, and high amplitude H-reflexes). One possible explanation for this outcome is a reduced relevance of spinal cord mechanisms involved in the modulation of reflex excitability during an isometric moderate contraction. In fact, it is argued that alteration in the PSI level is responsible for the differences in reflex modulation between older and young adults during contraction above 30% MVC. Additionally, in line with the present results for the soleus muscle, the reflex modulation in an ankle dorsiflexor of the elderly below this level of contraction matches those observed in young adults (Klass et al., 2011).

During MVC there is a diminished Ia activity and an enhanced low-threshold group III and IV afferent input, both leading to a reduction in reflex excitability, but the descending excitatory drive seems to overcome those inhibitory effects (Löscher et al., 1996). Even with a reduced contraction level, as the one currently used, it seems reasonable to argue that the effects of the descending drive on MN membrane potential may also outweigh the modulation by other local networks (e.g., involved in PSI), being the major contributor to the increased reflex pathway excitability during a moderate voluntary contraction. The present experimental and simulated results are in consonance with this notion. Notwithstanding, this conclusion was reached by exclusion as neither pre or postsynaptic spinal cord modulatory mechanisms were incorporated in the computational model. An alternative explanation would be a complex interaction of spinal cord mechanisms of reflex modulation resulting in the same observed pattern. It is also important to bear in mind that the action of spinal cord pathways mediating either peripheral or segmental signaling is complex and depends on a variety of factors, such as the performance of motor tasks (Sirois et al., 2013). Indeed, Kido et al. (2004) showed a clear effect of aging on reflex modulation for subjects in standing position. Future investigations considering the whole ascending limb of the RC, using more comprehensive simulations and/or experimental procedures (e.g., PSI evaluation), could provide a cleared view of the ensuing spinal cord neurophysiology adaptations during a moderate isometric voluntary muscle contraction for both populations.

### Methodological considerations

A recurrent issue in H-reflex studies is the maintenance of a comparable M-wave amplitude across different motor tasks (Capaday and Stein, 1986; Frigon et al., 2007). In the present work, the comparison between reflex amplitudes was allowed through the normalization by Mmax (i.e., the corresponding amplitude parameters from the sigmoidal fit), as well as the stimulus intensities by the current that evoked a M wave with amplitude of 50% Mmax (Klimstra and Zehr, 2008). This procedure enables the comparison of reflex excitability for the respective M-wave amplitude or the same input (nearly the same Ia afferent volley) in different conditions.

However, even during an isometric contraction, the amplitude of the Mmax might vary due to muscle fiber shortening, leading to alterations in the relation of the muscle and the surface EMG electrode (Frigon et al., 2007). In fact, there is no consensus regarding the behavior of Mmax during contraction. Some authors found an increase in Mmax during contraction (Pensini and Martin, 2004; Frigon et al., 2007; Racinais et al., 2013), while others did not find any change (Duclay et al., 2008; Grosprêtre and Martin, 2012). The present results are in accordance with previous data showing increased Mmax with isometric muscle contraction probably due to geometric factors as suggested by Frigon et al. (2007). It seems that those factors can override the hypoexcitability found in motor axons during contraction (Vagg et al., 1998). Therefore, we reinforce the recommendation for referencing H-reflex amplitudes to the respective Mmax in different conditions (Pensini and Martin, 2004; Racinais et al., 2013).

There is also some controversy around the effect of aging on Mmax amplitude, with a few studies reporting that the Mmax does not change with age (Kawashima et al., 2004; Nakazawa et al., 2006; Piirainen et al., 2012), while others showed a decreased Mmax amplitude for the elderly (Chalmers and Knutzen, 2002; Scaglioni et al., 2002, 2003; Baudry et al., 2015). In the current work, the absolute values of Mmax amplitude at rest for both groups were similar to those found elsewhere (Kawashima et al., 2004; Piirainen et al., 2012), but were significantly reduced (42.1%) in the elderly as compared to young adults, in consonance with previous findings (57.3% of reduction in the elderly group) (Scaglioni et al., 2003). The significant differences in Mmax amplitudes between both groups probably rely on either the loss of large MNs (Luff, 1998; Raffalt et al., 2015) or changes in muscle tissue leading to a slower action potential propagation along the muscle fibers in older participants (Scaglioni et al., 2003). Both phenomena could affect the H-reflex amplitude as well. Therefore, it is unlikely that the significant reduction in Mmax in the senior group would mask possible between-groups differences in H-reflex parameters from the ascending limb of the RC. Albeit there was absence of significant differences in some of the previous studies, it was observed a tendency to increase Mmax with contraction (Grosprêtre and Martin, 2012) and decrease it with age (Kawashima et al., 2004; Nakazawa et al., 2006; Piirainen et al., 2012). One possible explanation for the lack of significant differences in Mmax reported by those authors could be related to an increased variability of the amplitude values (which cannot be normalized), rendering the statistical test less sensitive.

We were unable to detect significant differences in Hmax parameter between groups (even considering the reduction of 23.5% for the elderly as compared to young adults), also in agreement with previous reports (reduction of 21.7%) (Scaglioni et al., 2003; Kawashima et al., 2004; Piirainen et al., 2012). Conversely, some researchers showed significant reduction in Hmax for the elderly (Sabbahi and Sedgwick, 1982; Angulo-Kinzler et al., 1998; Dewhurst et al., 2005; Baudry et al., 2015). The divergence among studies might stem from methodological aspects such as posture (siting versus prone position), characteristic of the population (such as level of physical activity), electrode configuration (monopolar versus bipolar) (Scaglioni et al., 2003), and the type of stimulation such as current (with control of skin impedance) versus voltage (which provide a less consistent nerve activation). The absence of a significant reduction in Hmax for the elderly might also be related to the lower averaged amplitude of Hmax for young subjects (in the present study, young values were closer to those from the elderly group). In fact, the values of Hmax from healthy young participants are variable across studies, e.g., being between 30 and 40% Mmax (Dewhurst et al., 2005), at around 58% Mmax (Scaglioni et al., 2003; Baudry et al., 2015), and at 37% Mmax as in the present work.

In contrast to our results, Grosprêtre and Martin (2012) reported absence of changes in H-reflex amplitudes at the ascending limb of the RC. It is possible that the lack of significant increments in reflex amplitude was due to the level of voluntary muscle contraction used by those authors (50% MVC). This level seems to be near to a limit for possible further increments in amplitude (Butler et al., 1993; Löscher et al., 1996). Similar result was achieved by Racinais et al. (2013), however, they showed differences for Hmax using 100% MVC. The method of sigmoidal fitting to the ascending limb of the RC currently used enables the normalization of stimulus intensities that are actually the input to the system. This represents an advantage compared to previous work, specially, when it is unclear how precise the (supra) maximal stimulus intensity (to generate Mmax) was defined. Therefore, with the present approach, we were able to defined parameters that represent reflexes with amplitudes near to the threshold, with 50% of the maximum, and with the highest amplitude, and compare them in different conditions always considering the same relative input.

## Conclusion

For a relatively weak isometric voluntary contraction used in a variety of works on spinal cord neurophysiology, the H-reflex amplitude increases irrespective of the sensory input (stimulus intensities at the ascending limb of the RC), and the general pattern of reflex modulation in the senior group does not differ from the young participants. Given the age-related changes in mechanisms of reflex modulation, one might surmise that the lack of differences between both groups relies on the overwhelming importance of descending pathway on the soleus MN pool. This argument is reinforced by the simulation results that showed similar facilitatory effect during a constant descending voluntary command. Notwithstanding, computational simulations with the addition of intrinsic spinal cord networks are advised to further explore the relevance of neurophysiological mechanisms of reflex modulation during contraction.

It is therefore suggested that experiments designed to study the ascending limb of the H-reflex RC in the elderly can be conducted without a possible confounding effect from a moderate voluntary contraction. The present research also showed that the Mmax amplitude actually increases with contractions regardless of the group considered, but the Mmax was typically lower in the elderly as compared to young participants. However, these changes do not affect the interpretation of the current results.

## Data availability statement

The raw data supporting the conclusions of this article will be made available by the authors, without undue reservation.

## Ethics statement

The studies involving human participants were reviewed and approved by Comitê de Ética da Faculdade de Ciências da Saúde da Universidade de Brasília. The patients/participants provided their written informed consent to participate in this study.

## Author contributions

RM, LE, GC, JC, and LB-F contributed to the conception and design of the study. RM, LE, and NR prepared the figures. RM and LB-F performed the statistical analysis. LB-F, NR, GC, JC, and LE performed the experiments. RM wrote the first draft of the manuscript. RM, LB-F, LE, and NR wrote sections of the manuscript. All authors organized the database and contributed to manuscript revision, read, and approved the submitted version.
